# Protective Effect of Rosamultin against H_2_O_2_-Induced Oxidative Stress and Apoptosis in H9c2 Cardiomyocytes

**DOI:** 10.1155/2018/8415610

**Published:** 2018-07-16

**Authors:** Ling Zhang, Yang Liu, Jian Yu Li, Ling Zhi Li, Yong Liang Zhang, Hai Ying Gong, Ying Cui

**Affiliations:** ^1^Department of Pharmacy, Logistics University of Chinese People's Armed Police Forces, Tianjin, China; ^2^Tianjin Key Laboratory for Prevention and Control of Occupational and Environmental Hazard, Tianjin, China

## Abstract

Rosamultin is one of the main active compounds isolated from *Potentilla anserina* L., which belongs to a triterpene compound. Few studies have examined the effect of rosamultin on oxidative stress and its molecular mechanism. The aim of this present study was to elucidate the protective effect of rosamultin on H_2_O_2_-induced oxidative damage and apoptosis in H9c2 cardiomyocytes and its mechanism. The results showed that the pretreatment of rosamultin not only increased cell viability but also reduced the release of LDH and CK. Rosamultin inhibited a H_2_O_2_-induced decrease in SOD, CAT, and GSH-Px activities and an increase in MDA content. Meanwhile, ROS level, intracellular (Ca^2+^) fluorescence intensity, and apoptosis rate in the rosamultin pretreated group were markedly decreased compared with the model group. Rosamultin pretreatment significantly reversed the morphological changes and attenuated H_2_O_2_-induced apoptosis. Western blot analysis showed that rosamultin enhanced the expression of Bcl-2 and pCryAB and downregulated the expression of Bax, Cyt-*c*, Caspase-3, and Caspase-9 expression. Additionally, rosamultin might activate PI3K/Akt signal pathways and CryAB relative factors. Therefore, we suggest that rosamultin could have the potential for treating H_2_O_2_-induced oxidative stress injury through its antioxidant and antiapoptosis effect.

## 1. Introduction

Oxidative stress plays a crucial role in the pathogenesis of various cardiovascular diseases such as heart failure, myocardial ischemia-reperfusion injury, cardiomyopathy, hypertension, atherosclerosis, metabolic syndrome, and atrial fibrillation [1–3]. Oxidative stress leads to the overproduction of ROS, which is an important event in the development of cardiovascular diseases [4]. Excessive ROS cause significant damage to myocardial cells, which can damage the oxidation-antioxidant equilibrium system [5]. Moreover, further development of the damage can lead to apoptosis [6]. Oxidative stress and apoptosis play important roles in the development of cardiovascular diseases [7]. Alleviating oxidative stress and/or a direct intervention on the inhibition of apoptotic pathways could provide potential molecular targets for therapeutic treatments [8–9].

**Figure 1:**
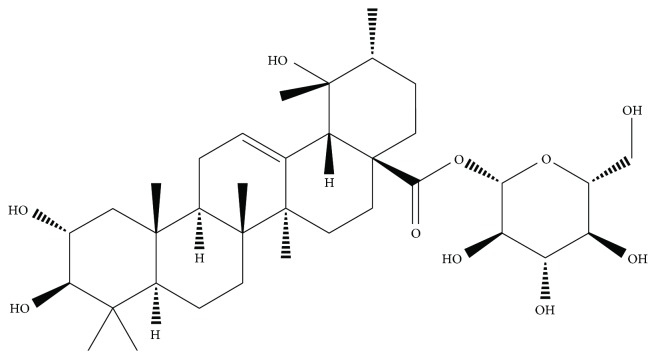
Chemical structure of rosamultin. Molecular formula: C_36_H_58_O_10_; molecular weight: 650.84.

It is very meaningful to discover and develop a novel natural product for the prevention and treatment of heart diseases caused by oxidative stress [10]. *Potentilla anserina* L. is a medicinal herb of the genus *Potentilla* in the family Rosaceae, which is widely distributed in western China. For thousands of years, this Tibetan traditional medicine has been widely used in its therapeutic properties [11–14]. Our previous pharmacological studies indicated that the n-butanol extract of *Potentilla anserina* L. had an obvious protective effect on cardiomyocytes by improving the scavenging ability of oxygen free radicals and inhibiting oxide injury to the membrane. Triterpenes are one of the major constituents of *Potentilla anserina* L., making it a good antioxidant. Rosamultin is a natural product from the subterranean root of the *Potentilla anserina* L. plant (Figure 1). Few studies have examined the effect of rosamultin on H_2_O_2_-induced oxidative stress injury in H9c2 cells and its molecular mechanism. Therefore, we investigated whether rosamultin exerted a protective effect by regulating the cryAB and PI3K/Akt signal pathway, further acting on apoptosis factors such as Bcl-2, Bax, Cyt-*c*, and Caspase-3, and Caspase-9 levels in the H9c2 cardiomyocyte.

## 2. Materials and Methods

### 2.1. Materials and Reagents

Rosamultin was purchased from Wuhu Delta Medical Technology Co. Ltd. (purity > 99%, Anhui, China), and dissolved in DMEM (without FBS). Cell culture materials were purchased from Thermo Fisher Scientific Inc. (Waltham, MA, USA). Biochemical detection kits were purchased from the Nanjing Jiancheng Bioengineering Institute. Antibodies were purchased from Abcam plc. or Protech Systems Co. Ltd. General laboratory reagents were purchased from Sigma-Aldrich (St. Louis, MO, USA).

### 2.2. Cell Culture and Treatment

The H9c2 cells derived from rat embryonic cardiomyocytes were obtained from the American Type Culture Collection. Cells were stored in 10% FBS and 1% penicillin/streptomycin DMEM under an atmosphere of 95% air and 5% CO_2_ at 37°C. The cells were seeded on glass coverslips or plastic wells 2 days before experiments to achieve a confluence of ~90%. Then, the oxidative stress model was established by exposure to a 200 *μ*M H_2_O_2_ solution in DMEM without FBS for 3 h.

### 2.3. Cell Viability Assay

Cell viability was examined by the MTT assay. H9c2 cells were plated in a 96-well plate at 1 × 10^5^ cells/well. After adhering overnight, H9c2 cells were incubated with rosamultin for 24 h except for the control group receiving the medium instead. Then, the H9c2 cells were treated with 200 *μ*M H_2_O_2_ for 3 h. After that, the cells were incubated with MTT (0.5 mg/ml) at 37°C for 4 h. Finally, the violet crystals were dissolved with 150 *μ*l DMSO after the medium was removed. Absorbance of each culture well was examined with a microplate reader at a wavelength of 490 nm.

### 2.4. Biochemical Analysis of H9c2 Cells

The H9c2 cells were seeded in a 24-well plate at a density of 1 × 10^5^ cell/ml and treated as described above. 100 *μ*l of the culture supernatant per well was collected, and the LDH and CK activities were measured with detection kits. LDH and CK activities were determined by the colorimetric method. Then, H9c2 cells were washed with cold PBS, and centrifuged at 300 ×g for 10 min. The supernatant was then removed. The precipitate obtained through centrifugation was crushed by ultrasonic wave, and the cell lysates were resuspended. SOD, GSH-Px, CAT, and MDA were determined with a microplate reader according to the protocol of the detection kit. GSH-Px activity was measured by the colorimetric method. SOD activity was detected by the xanthine oxidase method. MDA content was measured by the thiobarbituric acid method. CAT activity was measured by the visible spectrophotometer method. Protein content was measured with the BCA Bradford protein assay.

### 2.5. Detection of Intracellular ROS

The intracellular ROS content was determined by the 2′,7′-dichlorofluorescin diacetate (DCFH-DA) method. Briefly, the H9c2 cells were incubated with 10 mM of DCFH-DA for 1 h at 37°C. After washing the extracellular DCFH-DA, the fluorescence intensity was determined by a flow cytometer. The excitation wavelength and emission wavelength were 488 nm and 525 nm, respectively. The content of intracellular ROS was expressed by the percentage of the control group.

### 2.6. Detection of Intracellular (Ca^2+^)

Intracellular Ca^2+^ flux was detected by using Fluo-3/AM as the fluorescent indicator. Briefly, cells were resuspended and incubated with a final concentration of 5 mM Fluo-3/AM dye for 15 min at 37°C. The fluorescence intensity of Ca^2+^ was photographed with LSCM.

### 2.7. Nuclear Staining

*For Hoechst-33342/PI staining*, briefly, the H9c2 cells were collected and washed twice with cold PBS. Then, the H9c2 cells were stained with fluorescent dyes. In brief, Hoechst-33342 and PI solution were added and incubated for 15 min at 37°C in the dark. Finally, the H9c2 cells were photographed by a fluorescent microscope. *For DAPI staining*, the H9c2 cells were harvested and washed twice with cold PBS, after which cells were fixed with 4% polyoxymethylene for 20 min. Then, cells were rinsed with PBS, and incubated with 1 *μ*M DAPI for 15 min at 37°C. Following staining, cells were rinsed and photographed using a fluorescent microscope.

### 2.8. Cell Apoptosis Assay

The Annexin V and PI fluorescein staining kits were used to determine apoptosis rate. The H9c2 cells were treated as previously described and collected with 0.25% trypsin. Then, cells were resuspended in 400 *μ*l of binding buffer solution and incubated with Annexin V/PI solution for 15 min in the dark. Apoptosis rate was detected by flow cytometry.

### 2.9. Western Blot Analysis

Total protein from H9c2 cells were prepared with RIPA lysing buffer. 20 *μ*g of the sample proteins of different groups were separated with 10% SDS-PAGE and transferred onto PVDF membranes. Membranes were incubated with a primary antibody, and followed by the incubation of the anti-rabbit IgG secondary antibody. Protein expression was detected with an enhanced chemiluminescence detection kit. *β*-Actin served as an internal control. The antibodies include Akt (1 : 1000), pAkt (1 : 1000), cryAB (1 : 1000), pCryAB (1 : 1500), Bcl-2 (1 : 1000), Bax (1 : 1000), Caspase-3 (1 : 1000), Caspase-9 (1 : 1000), Cyt-*c* (1 : 1000), and *β*-actin (1 : 4000).

### 2.10. Statistical Analysis

Data are expressed as means ± S.E.M. Statistical analyses were conducted by one-way analysis of variance (ANOVA) and the comparisons between groups were performed by Dunnett's test. A *P* value less than 0.05 was considered statistically significant.

## 3. Results

### 3.1. Effect of Rosamultin on Cell Viability

**Figure 2:**
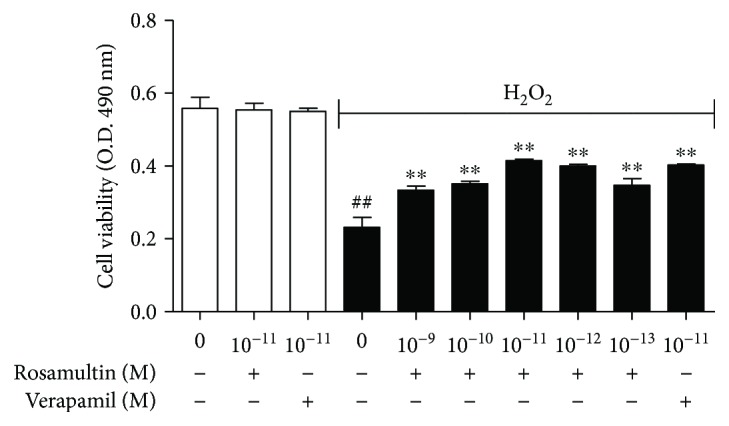
Effect of rosamultin on H9c2 cell viability after being subjected to H_2_O_2_-induced oxidative damage. H9c2 cells were preincubated in the presence and absence of rosamultin (10^−13^–10^−9^ M) for 24 h and then stimulated further with 200 *μ*M of H_2_O_2_ for an additional 3 h. After MTT was added, the violet crystals were dissolved with DMSO. Absorbance was measured with a microplate reader at 490 nm. Data are mean ± S.E.M. (*n* = 6). ^##^*P* < 0.01 versus the control group and ^∗∗^*P* < 0.01 versus the model group.

The H9c2 cells were incubated with rosamultin (0, 10^−9^, 10^−10^, 10^−11^, 10^−12^, and 10^−13^ M) for 24 h. Verapamil (10^−11^ M) served as the positive control drug. Rosamultin and verapamil caused no damage to cells compared with the control group (*P* > 0.05). However, the viability of H9c2 cells incubated with H_2_O_2_ (200 *μ*M) for 3 h decreased obviously compared with the control group (*P* < 0.01). The viability of cells pretreated with rosamultin (10^−9^, 10^−10^, 10^−11^, 10^−12^, and 10^−13^ M) for 24 h before exposure to H_2_O_2_ significantly increased compared with the model group (*P* < 0.01), demonstrating the protective effect of rosamultin on H9c2 cells from H_2_O_2_-induced oxidative stress damage (Figure 2).

### 3.2. Effect of Rosamultin on LDH and CK Activities

**Figure 3:**
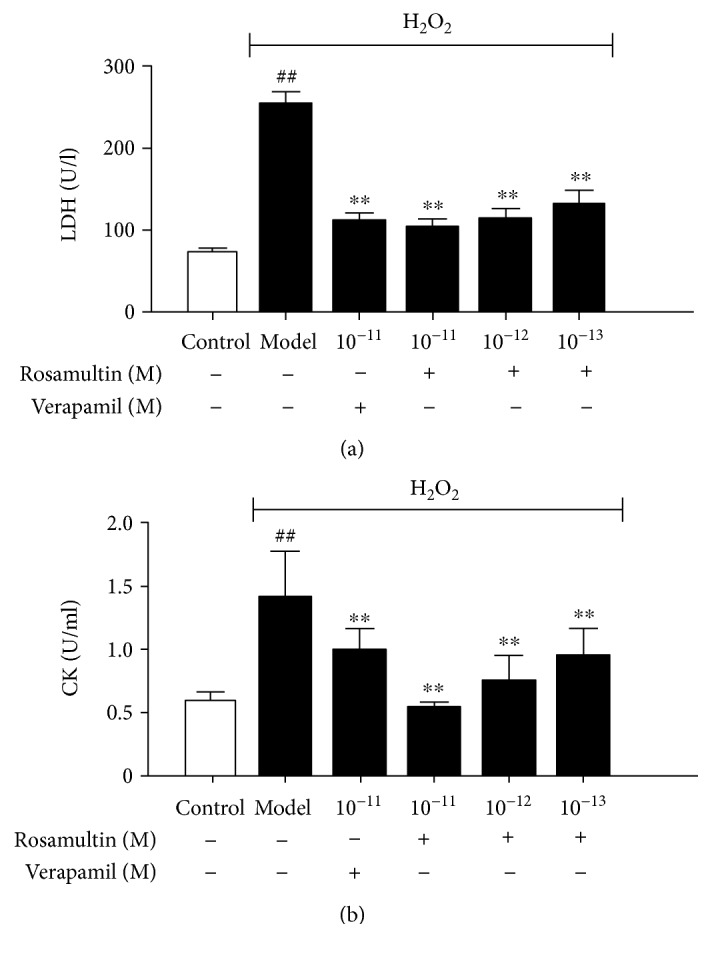
Effect of rosamultin on LDH and CK activities in the culture supernatant of H9c2 cardiomyocytes subjected to H_2_O_2_-induced oxidative damage. Data are mean ± S.E.M. (*n* = 6). ^##^*P* < 0.01 versus the control group and ^∗∗^*P* < 0.01 versus the model group.

To confirm whether rosamultin has the protective effects against H_2_O_2_-induced oxidative stress damage, we assayed the release of LDH and CK in different groups. The activity of LDH and CK in the culture medium was considered an indicator for cell membrane damage. The release of LDH and CK obviously increased in the model group compared with that in the control group (*P* < 0.01). However, the LDH and CK activities in the culture medium were significantly decreased after pretreatment with rosamultin or verapamil compared with that in the model group (*P* < 0.01) (Figure 3).

### 3.3. Effect of Rosamultin on Antioxidant Enzyme and Lipid Peroxidation

**Figure 4:**
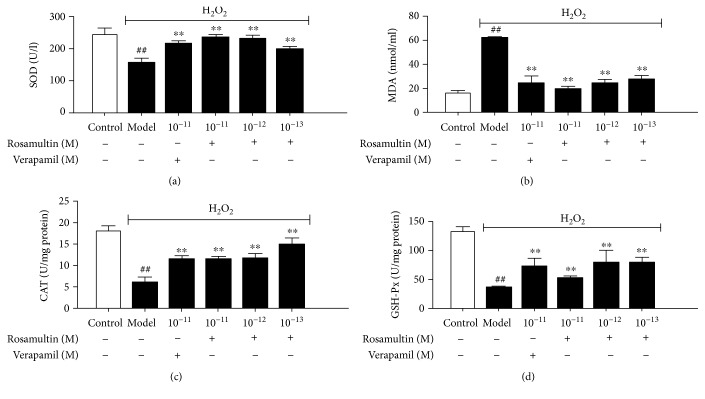
Effect of rosamultin on SOD, CAT, and GSH-Px activities and MDA content in the lysates of H9c2 cardiomyocytes subjected to H_2_O_2_-induced oxidative damage. Data are mean ± S.E.M. (*n* = 6). ^##^*P* < 0.01 versus the control group and ^∗∗^*P* < 0.01 versus the model group.

To determine whether rosamultin affects oxidative stress-related biochemical enzymes, the levels of antioxidant enzymes, such as SOD, CAT, GSH-Px, and the lipid peroxidation products, such as MDA, were assayed in H9c2 cell lysates. SOD, CAT, and GSH-Px activities were remarkably decreased while MDA production significantly increased in the model group compared with the control group (*P* < 0.01). Rosamultin pretreatment could decrease MDA production and increase the activities of SOD, CAT, and GSH-Px compared with that in the model group (*P* < 0.01) (Figure 4). The results confirmed that rosamultin had a strong antioxidant capacity *in vitro*.

### 3.4. Effects of Rosamultin on Intracellular ROS Generation

**Figure 5:**
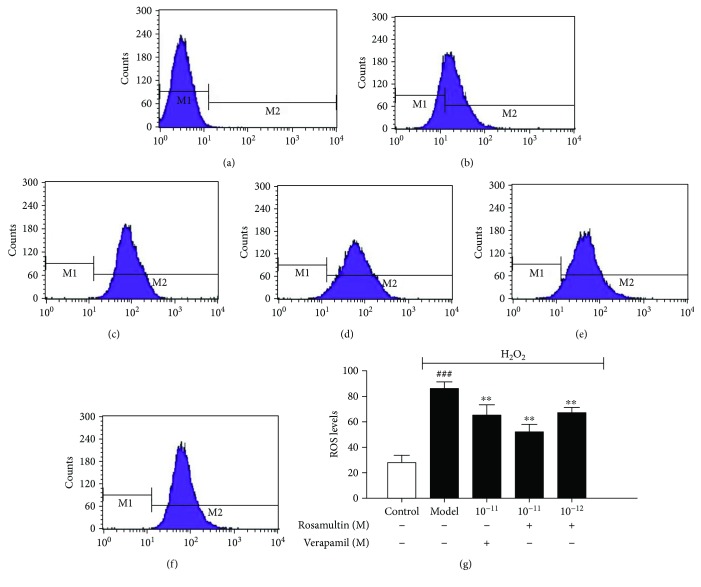
Effect of rosamultin on ROS levels of H9c2 cardiomyocytes that suffered H_2_O_2_-induced oxidative damage. Intracellular ROS levels were measured with the DCFH-DA assay. (a) Control without DCFH-DA, (b) control group, (c) model group, (d) verapamil 10^−11^ M group, (e) rosamultin 10^−11^ M group, and (f) rosamultin 10^−12^ M group. Data are mean ± S.E.M. (*n* = 6). ^##^*P* < 0.01 versus the control group and ^∗∗^*P* < 0.01 versus the model group.

When cells are exposed to H_2_O_2_, oxidative stress is accompanied with an increase in intracellular ROS levels. To determine whether rosamultin affects H_2_O_2_-induced ROS generation, the intracellular ROS level was determined with the DCFH-DA method. The ROS level in the model group increased significantly compared with the control group (*P* < 0.01). However, ROS levels in rosamultin pretreatment groups were significantly reduced (*P* < 0.01), suggesting that rosamultin is a potent cardioprotective agent against H_2_O_2_-induced oxidative stress (Figure 5).

### 3.5. Effects of Rosamultin on Cytosolic Ca^2+^

**Figure 6:**
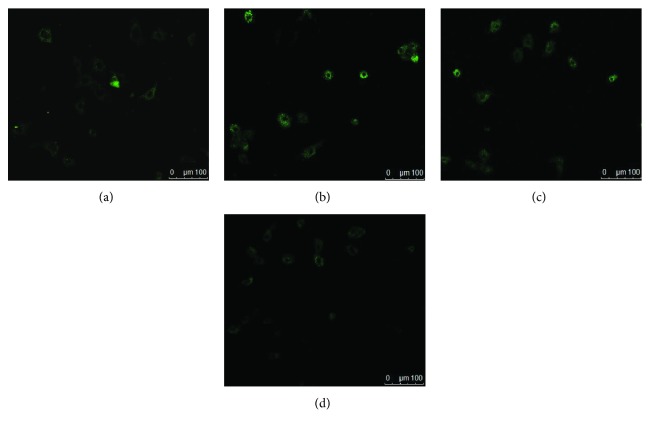
Intracellular Ca^2+^ flux was measured by Fluo-3/AM staining in H9c2. Cytosolic Ca^2+^ fluorescence intensity was photographed with LSCM (×200). (a) Control group, (b) model group, (c) rosamultin 10^−11^ M group, and (d) verapamil 10^−11^ M group.

Cytosolic calcium overload and the destruction of calcium homeostasis are thought to initiate myocardial injury. In the model group, we observed higher fluorescence intensity than in the control group. Furthermore, the cytosolic calcium fluorescence intensity in rosamultin pretreatment groups were weakened compared with the model group (Figure 6).

### 3.6. Morphological Observation of Apoptotic Myocytes by Hoechst 33342/PI Double Staining

**Figure 7:**
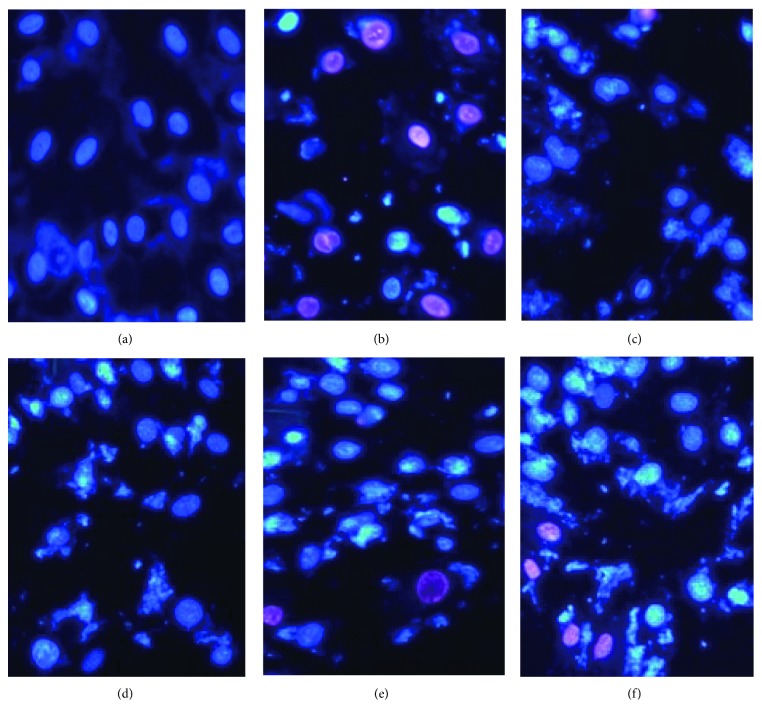
A double-fluorescent staining kit was used for the detection of the cell cycle and cell necrosis. After Hoechst 33342/PI fluorescent staining, the normal cells, apoptotic cells, and necrotic cells can be distinguished from the flow cytometer (×200). (a) Control group, (b) model group, (c) verapamil 10^−11^ M group, (d) rosamultin 10^−11^ M group, (e) rosamultin 10^−12^ M group, and (f) rosamultin 10^−13^ M group.

A double-fluorescent staining kit was used for the detection of the cell cycle and cell necrosis. After Hoechst 33342/PI staining, the normal, apoptotic, and necrotic cells can be distinguished by the flow cytometer. Normal cells with tropochrome on Hoechst 33342 are shown to have a weak blue and red fluorescence; apoptotic cells with chromatophilia are shown to have a strong blue and weak red fluorescence; and necrotic cells with chromatophilia on PI are shown to have a weak blue and strong red fluorescence. The normal cells had uniformly dispersed chromatin and intact membranes. After cells were exposed to H_2_O_2_, we observed that apoptotic cells with nuclear shrinkage, chromatin condensation, and fragmentation increased, and a few apoptotic bodies had also appeared in the model group. However, the apoptotic cells in rosamultin pretreatment groups were obviously reduced compared with the model group, and the appearance of nuclear condensation and fragmentation were obviously reduced (Figure 7).

### 3.7. Morphological Assessment of Apoptotic Myocytes by DAPI Staining

**Figure 8:**
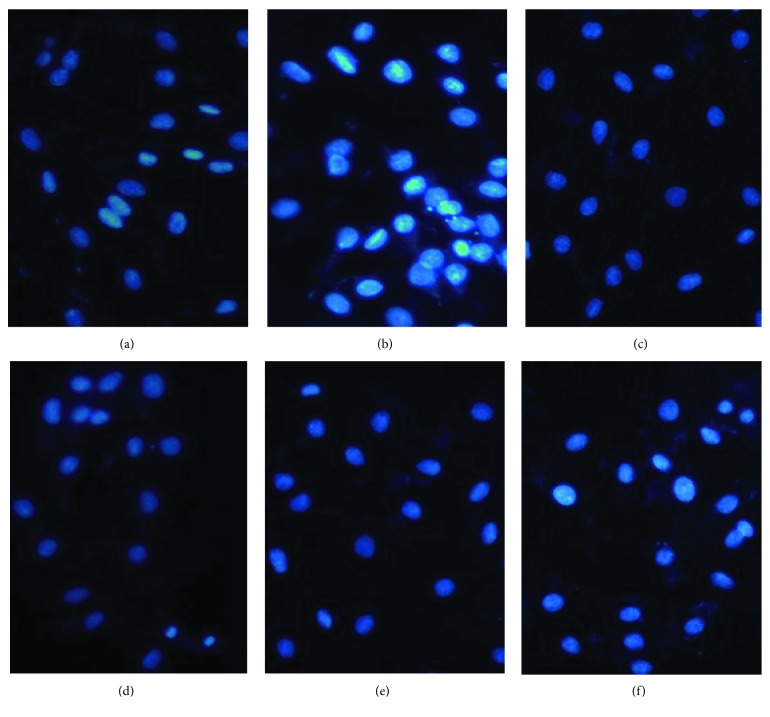
DAPI, a fluorescent dye, can penetrate the cell membrane and bind to the double-stranded DNA in the nucleus. The morphological changes were observed under a fluorescence microscope (×200). (a) Control group, (b) model group, (c) verapamil 10^−11^ M group, (d) rosamultin 10^−11^ M group, (e) rosamultin 10^−12^ M group, and (f) rosamultin 10^−13^ M group.

To clarify whether rosamultin protects H9c2 cells by antiapoptosis, we detected apoptotic nuclei and DNA fragmentation in H_2_O_2_-treated cells with or without rosamultin. DAPI is a widely used fluorescent dye in the apoptosis assay, which can penetrate membranes, bind to the double-stranded DNA in the nucleus, and play the role of labeling. DNA condensation is a typical characteristic in apoptosis. Under a fluorescence microscope, we could see the morphological change of the nucleus. In the control group, the shape of cardiomyocytes were round with normal organelles and intact cell membranes, and a light blue fluorescence was observed. After incubation with H_2_O_2_, cardiomyocyte shrinkage, pyknosis, and lobulated karyorrhexis, a bright blue fluorescence was seen. However, the above morphological changes in the rosamultin pretreatment group were obviously improved (Figure 8).

### 3.8. Effects of Rosamultin on Cell Apoptosis

**Figure 9:**
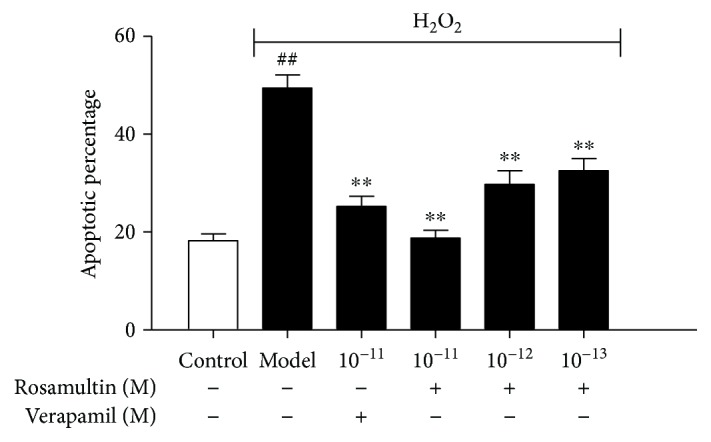
Effect of rosamultin on the apoptosis rate of cardiomyocytes that suffered H_2_O_2_-induced oxidative damage. The apoptotic rate was determined by Annexin V/PI staining, and samples were analyzed by a flow cytometer. Data are mean ± S.E.M. (*n* = 3). ^##^*P* < 0.01 versus the control group and ^∗∗^*P* < 0.01 versus the model group.

Furthermore, we also detected the apoptotic rate by Annexin V/PI staining. As shown in Figure 9, the cell apoptotic rate in the model group was higher than that in the control group (*P* < 0.01). Pretreatment with rosamultin remarkably decreased the apoptotic rate compared with that of the model group (*P* < 0.01).

### 3.9. Mechanism of Rosamultin against H_2_O_2_-Induced Oxidative Stress

**Figure 10:**
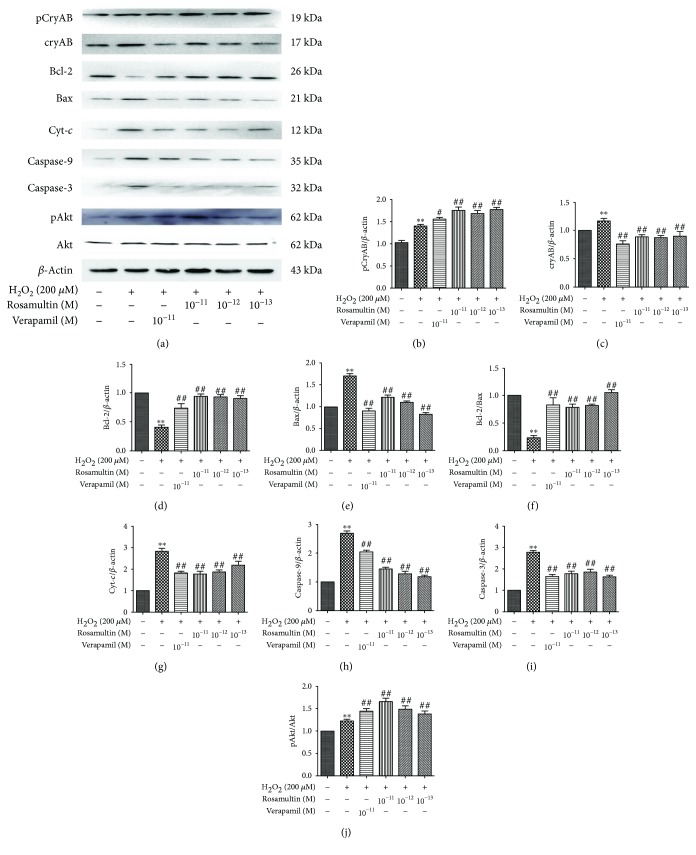
Effects of rosamultin on protein levels of apoptotic-related molecules in H9c2 cells that suffered H_2_O_2_-induced oxidative damage. (a) Representative Western blots, (b–i): pCryAB, CryAB, Bcl-2, Bax, Bcl-2/Bax, Cyt-*c*, Caspases-9, Caspases-3, and pAkt/Akt protein expression. Data are mean ± S.E.M. (*n* = 3). ^∗∗^*P* < 0.01 versus the control group, ^##^*P* < 0.01 versus the model group, and ^#^*P* < 0.05 versus the model group.

**Figure 11:**
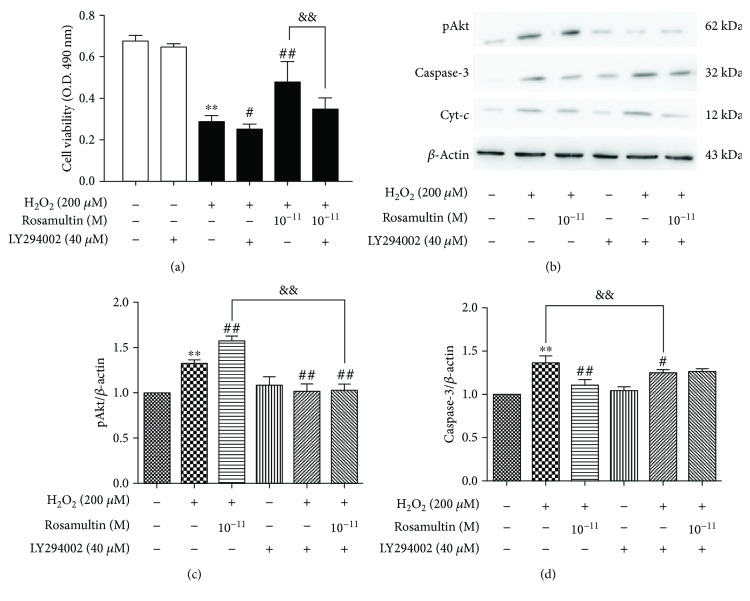
The effect of rosamultin on the PI3/Akt signaling pathway in H_2_O_2_-treated H9c2 cells. Cells were precultured in the presence or absence of 40 *μ*M LY294002, a specific inhibitor of PI3K, and incubated with or without rosamultin for 24 h; after that, cells were stimulated further with 200 *μ*M H_2_O_2_ for an additional 3 h. (a) Effect of rosamultin on H_2_O_2_-induced cell damage in the presence and absence of a PI3K inhibitor. Cell vitality was measured by MTT assay. (b) Representative Western blots. (c) pAkt protein expression. (d) Caspase-3 protein expression. Data are mean ± S.E.M. (*n* = 3). ^∗∗^*P* < 0.01 versus the control group, ^##^*P* < 0.01 versus the model group, ^#^*P* < 0.05 versus the model group, and ^&&^*P* < 0.01 versus the rosamultin pretreatment group.

Western blot was applied to evaluate the molecular mechanism of rosamultin under H_2_O_2_-induced oxidative stress. The phosphatidylinositol-3-kinase (PI3K)/Akt pathway and cryAB are involved in the regulation of apoptosis in H9c2 cells. Therefore, we confirmed whether the PI3K/Akt signal pathway and cryAB were regulated by rosamultin under H_2_O_2_-induced apoptosis. As shown in Figure 10, H_2_O_2_ treatment decreased Bcl-2, Bcl-2/Bax, and pAkt expression, but increased cryAB, Bax, Cyt-*c*, Caspase-9, and Caspase-3 expression, compared with the control group; however, these changes in the model group were reversed by rosamultin. The expression of Akt had no significant changes between the groups. The level of phosphorylation cryAB (pCryAB) obviously increased in both the model group and the rosamultin pretreatment groups, but pCryAB expression in the model group was lower than that of the rosamultin pretreatment group. To confirm whether the PI3K/Akt signaling pathway was involved in the antiapoptosis function of rosamultin, H9c2 cells were preincubated with or without the PI3K/Akt specificity inhibitor LY294002. As a result, LY294002 abolished the effect of rosamultin on the PI3K/Akt signaling pathway, such that LY294002 decreased cell viability and increased Caspase-3 and Cyt-*c* apoptosis-related protein level (Figure 11).

## 4. Discussion

*Potentilla anserina* L. is a medicinal herb of the genus *Potentilla* in the Rosaceae family, which is widely distributed in western China, such as in Tibet, Gansu, and the Qinghai province. For thousands of years, this Tibetan medicine has been widely used for its therapeutic properties [11–14]. The major constituents of *Potentilla anserina* L. are flavonoids, triterpenes, polyphenols, and polysaccharides [11, 15-16]. Our previous pharmacological studies indicated that the n-butanol extract of *Potentilla anserina* L. showed a significantly protective effect on cardiac myocytes by improving their ability to scavenge oxygen free radicals and inhibiting oxidative injury to the membrane. Numerous in vitro and in vivo studies have revealed the multidirectional properties of triterpenes, including anti-cancer, antioxidant, anti-inflammatory, anti-atherosclerotic and antiviral [17]. Rosamultin is a natural product extracted from the root of *Potentilla anserina* L. Few reports about its pharmacological activity have been reported. In the present study, we studied the effect and molecular mechanism of rosamultin on H_2_O_2_-induced oxidative stress and apoptosis in H9c2 cells.

Oxidative stress plays an important role in the pathogenesis of various cardiovascular diseases. However, the effects of rosamultin on oxidative stress injury in cardiomyocytes have not been elucidated. Myocardial apoptosis during oxidative stress damage is frequently associated with excessive ROS production [18–19]. Therefore, inhibiting oxidative damage and apoptosis induced by oxidative stress is an important intervention strategy for cardiovascular diseases. We utilized H9c2 cardiomyocytes, incubated with H_2_O_2_ to establish an oxidative stress damage model for examining the protective effect and mechanisms of rosamultin. We used verapamil, which is a calcium channel antagonist, as a positive control.

We determined cell viability by the MTT method. After 3 h of incubation with H_2_O_2_, cell viability was decreased in the model group; but pretreated with rosamultin, the proliferation activity was observably increased. The concentrations of rosamultin chosen in the experiment were without toxic effect. LDH and CK are indicators of the integrity of the cell cytoplasmic membrane, which cannot penetrate the membrane under the physiological state; but when cell membrane damage occurs, LDH and CK are leaked from the intracellular endochylema to the extracellular matrix [20]. The results showed that LDH and CK activity in the rosamultin pretreatment group remarkably decreased compared with the model group, implying that rosamultin has the ability to protect cardiomyocytes from H_2_O_2_-induced membrane damage.

Oxygen is a critically essential substance for cardiomyocytes, usually accompanied with potentially dangerous ROS generated by mitochondrial respiration [21]. ROS could interact with biological macromolecules, such as nucleic acids, proteins, and lipids, thereby finally causing cell dysfunction, manifested as cytotoxicity, intracellular ATP depletion, and Ca^2+^ overload [22]. The balance between an oxidant and an antioxidant plays a vital role in maintaining normal biological function. Exogenous stimulation can break the balance, cause excessive accumulation of ROS *in vivo*, and induce many diseases [23]. Cellular antioxidant enzymes, such as SOD, CAT, and GSH-Px could decrease intracellular ROS content [24, 25], although the lipid peroxidation products can indirectly reflect the generation of ROS under oxidative stress [26]. Furthermore, we examined whether rosamultin had an antioxidative activity under oxidative stress. We measured MDA and ROS content and the activity of cellular antioxidants, including SOD, CAT, and GSH-Px. The results clearly showed that rosamultin pretreatment could reduce MDA and ROS generation and improve antioxidant enzyme activity. The above results suggested that the effect of rosamultin might be partly due to improving the balance of the oxidant and antioxidant system.

The calcium ion is mainly distributed in the endoplasmic reticulum and mitochondria and plays a vital role in maintaining normal cell function. It is generally believed that the occurrence of calcium overload and generation of excessive ROS has a reciprocal causality in the oxidative stress process [27]. The excessive ROS produced by oxidative stress could damage membrane structure, increase membrane permeability, and cause a large Ca^2+^ influx outside the cell, resulting in intracellular calcium overload. Calcium overload will further aggravate the production of ROS [28]. Based on the aforementioned analysis, rosamultin significantly decreased the fluorescence intensity, indicating that rosamultin might inhibit H_2_O_2_-induced calcium overload in cardiomyocytes.

Apoptosis is a programed and active mode of a cell death pathway, regulated by its own genes in a normal physiological or pathological environment [29]. H9c2 cells treated with H_2_O_2_ could promote the apoptosis of cells. H_2_O_2_-induced apoptosis in the present study was evidenced by morphological change, which was observed by Hoechst 33324/PI and DAPI staining. The characteristics of apoptosis are cell shrinkage, DNA fragmentation, chromatin condensation, and an apoptotic body [30]. The results strongly showed that rosamultin might play a protective role against H_2_O_2_-induced apoptosis.

Because of its potential relativity to oxidative stress damage and cell apoptosis, our aim was to identify possible pathways, in which H_2_O_2_-induced damage may be involved. Oxidative stress damage leads to the disruption of the mitochondrial membrane, and then result in an MPTP opening, which is an important factor in the mitochondrial apoptotic pathway [31]. An MPTP opening further leads to the shift of Cyt-*c* from the mitochondria to the cytoplasm. Cyt-*c* could combine with Apaf-1 and procaspase-9 to generate complex apoptotic bodies, which activates a Caspase-3 cascade reaction and induces apoptosis [32, 33]. To examine whether rosamultin participated in the mitochondrial apoptotic pathway, we determined protein changes involved in mitochondrial injury by Western blot [34]. The results showed that H_2_O_2_ could increase Bax, Cyt-*c*, Caspase-9, and Caspase-3 expression, and inhibit Bcl-2 synthesis, which results in an imbalance between Bcl-2 and Bax. Finally, the cells got involved in the program of apoptosis. Rosamultin pretreatment could inhibit apoptosis by decreasing Bax, Cyt-*c*, Caspase-9, and Caspase-3 expression, and increasing Bcl-2 synthesis.

Various mechanisms have been reported to explicate myocardial damage during oxidative stress [35, 36]. ROS causes cell injury directly or through intermediate products in the PI3K/Akt signaling pathway [37, 38]. In addition, activation of the PI3K/Akt pathway may be related to the Bcl-2 family gene [39]. The PI3K/Akt pathway activation could protect cardiomyocytes against apoptosis [40]. In the present study, Akt mainly exists in an activation and phosphorylation form under oxidative stress. Therefore, the activation of the PI3K/Akt pathway seems to be the cause of rosamultin's antiapoptotic effect. LY294002, as a specific inhibitor of PI3K and an upstream activator of Akt, eliminated the protective action of rosamultin. On the other hand, Xu et al. [41] reported that cryAB overexpression could protect H9c2 cells from H_2_O_2_-induced apoptosis, such as decreasing mitochondria Cyt-*c* release and increasing Bcl-2 expression [42]. The CryAB protein shows rapid phosphorylation, which regulates its activity in response to multiple stimuli, including oxidative stress. In our study, after treatment with H_2_O_2_, the levels of cryAB and pCryAB in cardiomyocytes were significantly upregulated, suggesting that when oxidative damage occurs, cardiomyocytes initiate stress responses to cope with apoptosis. CryAB was phosphorylated at three different serine sites, including 19, 45, and 59 serine residues. Therefore, the different cryAB phosphorylation sites have different functions. Ser19 and ser45 have strong inhibitory effects alone or in combination, while ser59 does not have an inhibitory effect, and could offset the inhibitory effect of monophosphorimide in ser19 and ser45. The phosphorylation of ser19 and ser45 alone or in combination resulted in a significant decrease in cryAB chaperone activity, whereas the monophosphorimide in ser59 had no such effect [43]. After being pretreated with rosamultin, the level of cryAB decreased, and the level of pCryAB S59 expression increased. It was presumed that these may be closely linked to the phosphorylated expression of cryAB S59, which resulted in incremental cryAB chaperone activity and enhanced regulatory activity of pCryAB S59. This indicates that rosamultin might play an antiapoptotic role by promoting cryAB S59 phosphorylation. However, the effect of cryAB and pCryAB on the PI3K/AKT pathway remains to be further studied.

## 5. Conclusion

In conclusion, the present study definitely illustrated that rosamultin protected H9c2 cardiomyocytes from H_2_O_2_-induced oxidative stress and apoptosis by reducing the level of Bax, Cyt-*c*, Caspase-9, and Caspase-3, increasing the Bcl-2/Bax ratio and pCryAB S59 expression, reducing ROS and MDA production, inhibiting calcium overload, and improving antioxidant enzyme activity, such as SOD, CAT, and GSH-Px. Furthermore, the PI3K/Akt pathway activation also participated in the cardioprotective effect of rosamultin. In a word, rosamultin could have the potential for the prevention and treatment of H_2_O_2_-induced oxidative stress damage in cardiomyocytes through its antioxidant and antiapoptosis effects.

## Data Availability

All data generated or analyzed during this study are included in this article.

## Abbreviations

DMEM: Dulbecco's modified Eagle's media
DMSO: Dimethyl sulfoxide
FBS: Fetal bovine serum
MTT: 3-(4,5-Dimethyl-2-thiazolyl)-2,5-diphenyl-2-H-tetrazolium bromide
OD: Optical density
LDH: Lactic dehydrogenase
CK: Creatine kinase isoenzyme
SOD: Superoxide dismutase
GSH-Px: Glutathione peroxidase
CAT: Catalase
MDA: Malonic dialdehyde
ROS: Reactive oxygen species
LSCM: Laser scanning confocal microscope
SDS-PAGE: Sodium dodecyl sulfate-polydactyl amide gel electrophoresis
Akt: Protein kinase B
PI3K: Phosphatidyl inositol 3-kinase
cryAB: *α*B-crystallin
Cyt-*c*: Cytochrome *c*
Bcl-2: B-cell lymphoma-2
Bax: Bcl-2-associated X protein
Caspase-3: Cysteinyl aspartate-specific proteinase-3
Caspase-9: Cysteinyl aspartate-specific proteinase-9
MPTP: Mitochondrial permeability transition pore.
